# Physicians’ self-perceived preparedness for clinical supervision of medical students at university and non-university hospitals -results from a Swedish survey

**DOI:** 10.1186/s12909-023-04908-8

**Published:** 2023-12-04

**Authors:** Paul Pålsson, Erik Hulegårdh, Mats Wahlqvist, Silvana Naredi, Katarina Jood

**Affiliations:** 1 Department of Education, Region Västra Götaland, NU-hospital group, Trollhättan, 46185 Sweden; 2 Institute of Neuroscience and Physiology, Department of Clinical Neuroscience, The Sahlgrenska Academy, University of Gothenburg, Gothenburg, Sweden; 3 Department of Research, Region Västra Götaland, Sahlgrenska University Hospital, Development, Education and Innovation, Gothenburg, Sweden; 4 Department of Anaesthesiology and Intensive Care, Institute of Clinical Sciences, Sahlgrenska Academy, University of Gothenburg, Gothenburg, Sweden; 5 Department of Neurology, Region Västra Götaland, Sahlgrenska University Hospital, Gothenburg, Sweden

**Keywords:** Clinical supervisor, Supervision, Medical student, Faculty development, Learning objectives

## Abstract

**Background:**

The need for clinical placements outside traditional teaching hospitals for medical students is growing, both due to a decrease in hospital beds and the expansion of medical students. In this survey, distributed to supervisors at university and non-university hospitals, we investigated supervisors’ self-perceived preparedness for the training assignment and searched for factors associated with self-perceived pedagogical knowledge and familiarity with the students’ learning objectives.

**Methods:**

A pilot survey was developed using results from qualitative studies regarding clinical supervision of medical students and included questions on the supervisors’ education and preparation, if they were familiar with the students’ learning objectives, self-perceived pedagogical knowledge, and characteristics of the learning environment. The pilot survey was tested on a smaller group of supervisors. The results from the pilot survey were used to develop an e-survey that was distributed to all hospital employed physicians in Region Västra Götaland.

**Results:**

The survey was completed by 1732 physicians (response rate 43%). Among 517 respondents at the university hospital who reported activity as supervisor, 240 (46%) had attended preparatory supervisor training, 423 (82%) perceived enough pedagogical knowledge for the teaching assignment, and 391 (76%) reported familiarity with the learning objectives. The corresponding proportions at non-university hospitals were 159/485 (33%), 363/485 (75%), and 298/485 (61%), respectively (p ≤ .007 all through, compared to the university hospital). Perceiving that goal description and written information from the course management was sufficient for being able to complete the training assignment showed strong association with both self-perceived pedagogical knowledge and familiarity with the students’ learning objectives.

**Conclusions:**

We found consistent differences between university and non-university hospitals with respect to the supervisors’ self-perceived preparedness for the training assignment. Efforts to convey the learning objectives and support to clinical supervisors are crucial for supervision of students at non-university hospitals.

## Introduction

The number of hospital beds per capita is dropping globally [1]. Meanwhile, the number of medical students requiring clinical placement is stable or increasing. As an example, from 1990 to 2015, the number of hospital beds per capita in Sweden were reduced by 80% [2, 3]. At the same time, from 1997 to 2017, the number of medical students in Sweden increased by 70% [4]. More students and fewer available patients in inpatient care led all medical schools in Sweden to expand their clinical rotations to smaller hospitals, not previously involved in undergraduate medical education. Similar trends are seen in UK and USA where a growing number of smaller hospitals are involved as sites for undergraduate training of medical students [5].

A smaller hospital within a region, associated with a medical school and a university hospital and admitting medical students, can be defined as a *regional clinical campus* [6]. Establishing a regional clinical campus requires adaptation in several areas. First, to supply the physical environment with adequate workspace for students’ educational activities. Second, to educate and prepare colleagues and staff to participate in clinical teaching [7, 8]. Reports show large variation in supervisors clinical interaction with students, and varying quality of clinical supervision [9–11].

When a regional clinical campus is established, local physicians must adapt to a new role, namely as clinical supervisors to medical students. Clinical supervisors at non-university hospitals usually have less formal educational training [12]. Research regarding clinical supervision of medical students indicate that there is a need for physicians both to learn more about basic educational principles and to develop a self-image as teachers to adapt to their new role [13, 14]. Hence, university courses that address students’ clinical supervisors should aim to improve teaching skills and confidence in teaching [15].

In addition to being skilled in teaching, physicians at a clinical campus need to know what the students are expected to learn [16–19]. Constructive alignment is commonly used as a tool to devise learning activities based on the intended learning outcomes [20, 21]. In the context of clinical rotation, the learning objectives point out to both students and supervisors what knowledge, skills and attitudes students are expected to acquire, in order to pass. Typically, early stages of learning are focused on knowledge and comprehension where later stages focus on analysis and synthesis [20, 22]. It follows that clinical supervisors need to know their students learning objectives to be able to adjust to the students’ needs and facilitate learning efficiently [23–26].

To date, the literature on how physicians at regional campuses are prepared for clinical supervision of medical students is limited. In the present study, we build on previous qualitative research concerning clinical supervision of medical students, conducted at our medical school [27–30]. The results from the qualitative research formed the base for the survey used in this study, with the aim to search for (a) factors associated with clinical supervisors’ self-perceived pedagogical knowledge and (b) their familiarity with students’ learning objectives at their clinical placements. Supervisors at non-university hospital are compared with those at a university hospital.

## Methods

### Setting

The health care system in Sweden is publicly funded and is organized in the same way throughout the country. Sweden is divided in six larger regions served by seven tertiary care university hospitals responsible for all highly specialized care. Smaller hospitals in each region can transfer patients to their university hospital. There are seven medical schools in Sweden, each of them associated with one of the seven tertiary care university hospitals.

The study was performed in Region Västra Götaland, an area in the south-west of Sweden with 1.7 million inhabitants. Inpatient- and specialized health care is provided by Sahlgrenska University Hospital in Gothenburg and three large county hospitals and several smaller. The hospitals range in size from a small 90 bed hospital to a large 2300 bed tertiary care university hospital. All hospitals within the region are through an agreement between the Swedish state, Region Västra Götaland and University of Gothenburg affiliated with the medical school at Sahlgrenska Academy, University of Gothenburg and are through the agreement, required to assist with clinical supervision for medical students. Thus, students do clinical rotations at all hospitals and most primary care units in the region. At each hospital department, there is a physician responsible for the medical students who takes part in faculty meetings at the university. The frequency of visits from faculty to the hospital units vary between courses. There are no formal requirements or certifications for teaching medical students, and for most rotations there are no mandatory preparatory supervisor courses. However, all residents in Sweden take a mandatory course in pedagogy as part of their training. The clinical rotations are evaluated by anonymous student course evaluations.

### Study design

This survey was designed by the unit for medical education and clinical learning at Sahlgrenska Academy, University of Gothenburg, Sweden. The unit consists of clinical physicians with experience from clinical supervision and senior lecturers from the medical program. The objective of the unit was to evaluate and promote clinical supervision.

Previous qualitative research at Sahlgrenska Academy regarding clinical supervision of medical students was used as a starting point. Factors with potential impact on clinical supervisors’ conditions and preparedness for supervision such as training in medical education, size of student groups and knowledge of the students’ learning objectives had been identified in interviews with clinical supervisors and from analysis of free-text questions in smaller surveys [27–30].

Using these themes, we developed a quantitative pilot survey with questions on the supervisors’ education and preparation, if they were familiar with the students’ learning objectives, self-perceived pedagogical knowledge and characteristics of the learning environment. To further investigate the perspective of the clinical supervisors, the survey ended with an open free-text question; “What can facilitate your task as a clinical supervisor?”, not reported in this study. The pilot survey was sent to all potential clinical supervisors working in internal medicine. Total number of recipients was 545 of which 265 responded (48% response rate).

The results from the pilot survey were used to finalize a larger survey. The list with courses in supervision or pedagogy was updated. As emails were described as an important way to receive information by several respondents, a question on receiving emails was added. Finally, questions using Likert scales were changed from five to six steps to enable equal dichotomization for analysis. Table 1 shows the questions and the answer alternatives of the final survey we used in this study.

**Table 1:** Survey questions

Question	Answer options
**The supervisors’ clinical competence level and pedagogical training**	
I work as	Intern (or before/after internship)/resident/consultant
My speciality / future speciality is (for residents and consultants)	Several alternatives
I have participated in preparatory supervisor training^2^	Several alternatives and free text
During the past year I have clinically supervised / taught:	Students/Interns/Residents/Consultants
I have mainly supervised students from the course	Several alternatives
During the spring semester 2018, I supervised medical students from the Sahlgrenska Academy.	Yes / No
**The supervisors’ knowledge of the training assignment**	
I have received an e-mail with information from the course management about the course structure.	Yes/No/Don’t know
The course management has visited my clinic and described the training assignment.	Yes/No/Don’t know
I have read the syllabus or other written tutor information from the course management for the current course	Six-point Likert scale^1^
I am familiar with the current learning objectives of the clinical placement	Six-point Likert scale^1^
I feel that I have enough pedagogical knowledge to carry out my educational assignment	Six-point Likert scale^1^
I feel that goal description and written information from the course management is sufficient for me to be able to complete the training assignment	Six-point Likert scale^1^
I feel that the clinical placement is long enough for students to achieve their learning objectives	Six-point Likert scale^1^
I feel that the clinical placement has a content that allows students to achieve their learning goals	Six-point Likert scale^1^
**Supervisors’ prerequisites for clinical teaching**	
How many medical students do you normally supervise at the same time in clinical practice?	Free text for each location (ward / outpatient clinic / emergency department / operating theatre)
I feel that I get enough support from the person at the clinic who is responsible for the medical students	Six-point Likert scale^1^
The days when I supervise in clinical practice, my production assignment is reduced by	0%/25%/50%/75%/100%
I feel that I have enough time set aside for my educational assignment	Six-point Likert scale^1^
Does the clinic have regular meetings for the physicians where the educational assignment is discussed?	Yes/No/Don’t know
I feel that there is sufficient organized support for the supervisors at the clinical location at my clinic	Six-point Likert scale^1^

^1^Six point Likert scale, from strongly disagrees to strongly agrees. Dichotomised in disagrees (1–3), agrees (4–6)

^2^Yes stands for that the physician has completed any university course in supervision

#### Survey distribution

Email addresses to all physicians employed at the hospitals in Region Västra Götaland were obtained from the regional employee registry. Physicians working at units without clinical rotations of medical students were excluded. The survey was distributed digitally 2018-10-16, using the digital survey tool ESMAKER [31].

Two reminders were sent by e-mail and the survey was closed 2018-12-09. The Esmaker survey system works with completely anonymised recipients. This means that it is not possible to link survey responses to receiving e-mail addresses or to be able to see afterwards which of the recipients responded to the survey. The system ensures that only one response per e-mail address can be registered.

#### Ethics

The research was carried out in accordance with guidelines and regulations stipulated in the Declaration of Helsinki. The study protocol was reviewed by the Swedish Ethical Review Authority (Reference number 2021 − 00859). According to the assessment, formal ethical approval was deemed unnecessary according to the Swedish law in §§ 3–4 of the act concerning the Ethical Review of Research Involving Humans (SFS 2003:460). Participants received written information about the aims of the study together with the electronic survey, the information included the information that the results from the survey could be used in a scientific study. Participation was voluntary and given that the survey was completely anonymous, and no personal data was collected or analysed, the study is exempted from direct informed consent according to the Swedish law concerning personal data (PUL 1998:204 3 §).

### Statistics

Responses to the questions using a six-point Likert scale were dichotomized by merging categories strongly agree / agree / slightly agree to “agree” and strongly disagree / disagree / slightly disagree to “disagree” [32, 33]. Differences between university hospital and non-university hospital supervisors’ responses in the proportion responding “agree” were tested for statistically significance using the χ2 test.

Two survey items were chosen as outcome measures indicating that the supervisor was prepared for their teaching assignment: “I feel that I have enough pedagogical knowledge to carry out my educational assignment.” and “I am familiar with the current learning objectives of the clinical placement “. All other questions were considered as potential factors associated with the outcome measures.

To investigate the association between the potential factors and the outcome measures we used logistic regression to calculate odds ratio of each potential factor for the two outcomes separately. A p-value ≤ 0.05 was considered statistically significant.

## Results

The survey was distributed to 3920 physicians.1732 surveys were completed resulting in a response rate of 43%. Of those, 730 reported that they had not been active as clinical supervisors in the previous semester and were excluded. Thus, a total of 1002 respondents were included in the final analysis.

### Characteristics of the clinical supervisor

The demographics of the participating physicians are described in Table 2. Of the 1002 respondents, 54% were consultants and 48% were working at non-university hospitals in the region. Most respondents were physicians engaged in the faculty courses “internal medicine” and “surgery”. There was a higher proportion of supervisors before residency in the non-university hospitals.

**Table 2:** The supervisor’s clinical competence level and pedagogical training

Variable	All = 1002 n (%)	University hospital = 517 n (%)	Non-University hospital = 485 n (%)
*Clinical competence level*			
Before residency^1^	148 (15)	46 (9)	102 (21)
Resident	315 (31)	171 (34)	144 (30)
Consultant	539 (54)	300 (59)	239 (50)
*Hospital type*			
University hospital	517 (52)		
Non-university hospital	485 (48)		
*Clinical supervisor in course*			
Internal medicine	296 (30)	157 (31)	139 (29)
Surgery^2^	296 (30)	144 (28)	152 (32)
Obstetrics, gynaecology	94 (10)	42 (8)	52 (11)
Paediatrics and child psychiatry	77 (8)	35 (7)	42 (9)
Infectious diseases	45 (5)	21 (4)	24 (5)
Psychiatry	44 (5)	38 (7)	6 (1)
Neurology	28 (3)	20 (4)	8 (2)
Introduction to clinical practice^3^	28 (3)	9 (2)	19 (4)
Ophthalmology	26 (3)	10 (2)	16 (3)
Dermatology	23 (2)	16 (3)	7 (1)
Otorhinolaryngology	17 (2)	15 (3)	2 (< 1)
Clinical consultation skills	12 (1)	2 (< 1)	10 (2)

^1^Including internship and clinical practice before internship

^2^The course in surgery includes orthopedics, anaesthesia/intensive care and oncology

^3^The introduction to clinical practice can be done in any speciality

### Preparatory supervisor training

In total, 40% of respondents reported that they had attended preparatory supervisor training. The rate was significantly higher for supervisors working at the university hospital compared to those working at non-university hospitals (Table 3). One small subgroup, 12% of respondents, reported that they had attended two or more preparatory supervisor training courses.

#### Knowledge of the training assignment

Familiarity with the students learning objectives was reported by 69% of respondents and 78% reported that they felt that they had enough pedagogical knowledge to supervise. Both rates were significantly higher for physicians at university hospitals compared to physicians at non-university hospitals. Receiving emails from course management was reported by 42% of respondents and 29% reported that course management had visited their workplace. Again, both rates were significantly higher for physicians at university hospitals compared to physicians at non-university hospitals (Table 3).

**Table 3:** The supervisors’ pedagogical training and knowledge of the training assignment divided by university and non-university hospital

Question	All = 1002 n (%)	University hospital = 517 n (%)	Non-University hospital = 485 n (%)	
	Yes/Agree	Yes/Agree	Yes/Agree	p-value*
**Pedagogical training**				
I have participated in preparatory supervisor training	399 (40)	240 (46)	159 (33)	< 0.001
**The supervisor’s knowledge of the training assignment**				
I have received an e-mail with information from the course management about the course structure^1^	425 (42)	268 (52)	157 (32)	< 0.001
The course management has visited my clinic and described the training assignment^1^	287 (29)	195 (38)	92 (19)	< 0.001
I have read the syllabus or other written tutor information from the course management for the current course	488 (49)	280 (54)	208 (40)	< 0.001
I am familiar with the current learning objectives of the clinical placement^1^	689 (69)	391 (76)	298 (61)	< 0.001
I feel that I have enough pedagogical knowledge to carry out my educational assignment^1^	786 (78)	423 (82)	363 (75)	0.007
I feel that goal description and written information from the course management is sufficient for me to be able to complete the training assignment^1^	588 (59)	350 (68)	238 (46)	< 0.001
I feel that the clinical placement is long enough for students to achieve their learning objective.^1^	595 (59)	311 (60)	284 (55)	0.606
I feel that the clinical placement has a content that allows students to achieve their learning goal.^1^	285 (28)	136 (26)	149 (29)	0.121

^*^Difference in responses between doctors working at non-university hospitals and university hospital, χ2 test

^1^Original question in Likert format, Likert 1–3 assigned label “Disagree”, Likert 4–6 assigned label “Agree”

### Supervisors’ clinical workload and support

The number of students supervised at the same time per clinical supervisor, was slightly higher in university hospital compared to non-university hospitals. Most supervisors were responsible for one to two students at the same time. A majority (74%) of respondents reported that they had no reduction in clinical workload while supervising students and a minority (38%) perceived that they had sufficient time for their task. These proportions did not differ significantly between respondents from university and non-university hospitals (Table 4). Scheduled meetings focused on teaching and learning were reported by 23% with a significantly higher frequency in the university hospital compared to the non-university hospitals (Table 3).

**Table 4:** The supervisor’s prerequisites for clinical teaching divided by university and non-university hospital

Question	All = 1002 n (%)	University hospital = 517 n (%)	Non-University hospital = 485 n (%)	
	Yes/Agree	Yes/Agree	Yes/Agree	p-value*
I feel that I get enough support from the person at the clinic who is responsible for the medical students. ^1^	701 (70)	383 (74)	318 (62)	0.003
The days when I supervise in clinical practice, my production assignment is reduced^2^ (%Yes)	262 (27)	118 (23)	144 (30)	0.011
I feel that I have enough time set aside for my educational assignment. ^1^	380 (38)	190 (37)	190 (39)	0.429
Does the clinic have regular meetings for the physicians where the educational assignment is discussed?	232 (23)	133 (26)	99 (20)	0.046
I feel that there is sufficient organized support for the supervisors at the clinical location at my clinic. ^1^	440 (44)	209 (40)	231 (45)	0.021

^*^Difference in responses between doctors working at non-university hospitals and university hospital, χ2 test

^1^ Original question in Likert format, Likert 1–3 assigned label “Disagree”, Likert 4–6 assigned label “Agree”

^2^ Original question with alternatives included how much the workload was reduced (0%/25%/50%/75%/100%), any reduction of workload was categorized as “Yes”

### Factors associated with supervisors’ preparedness to teach

**Fig. 1:**
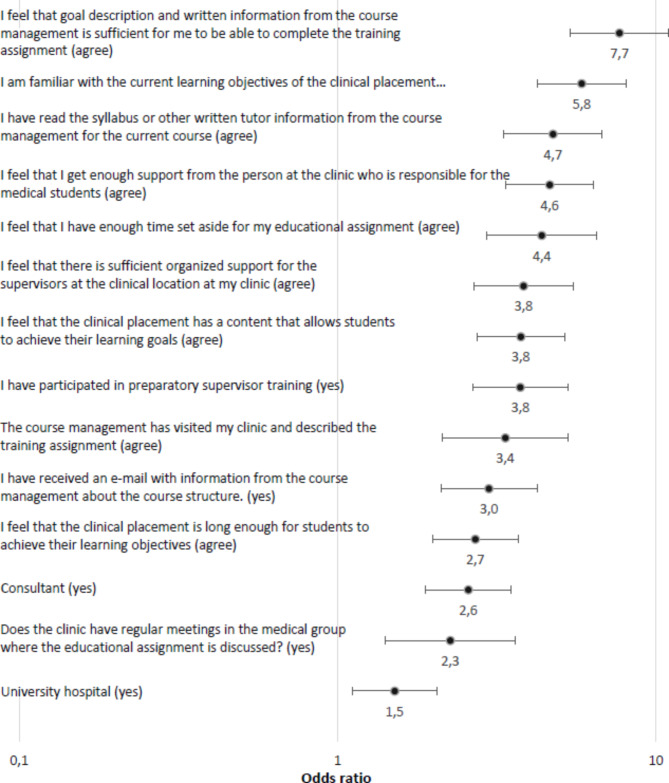
“I feel that I have enough pedagogical knowledge to carry out my educational assignment.” Odds ratios and 95% confidence intervals for variables with respondents reporting agree to the statement. Odds ratios with factor present (yes/agree) compared to factor not present, ranked by strength of association

**Fig. 2:**
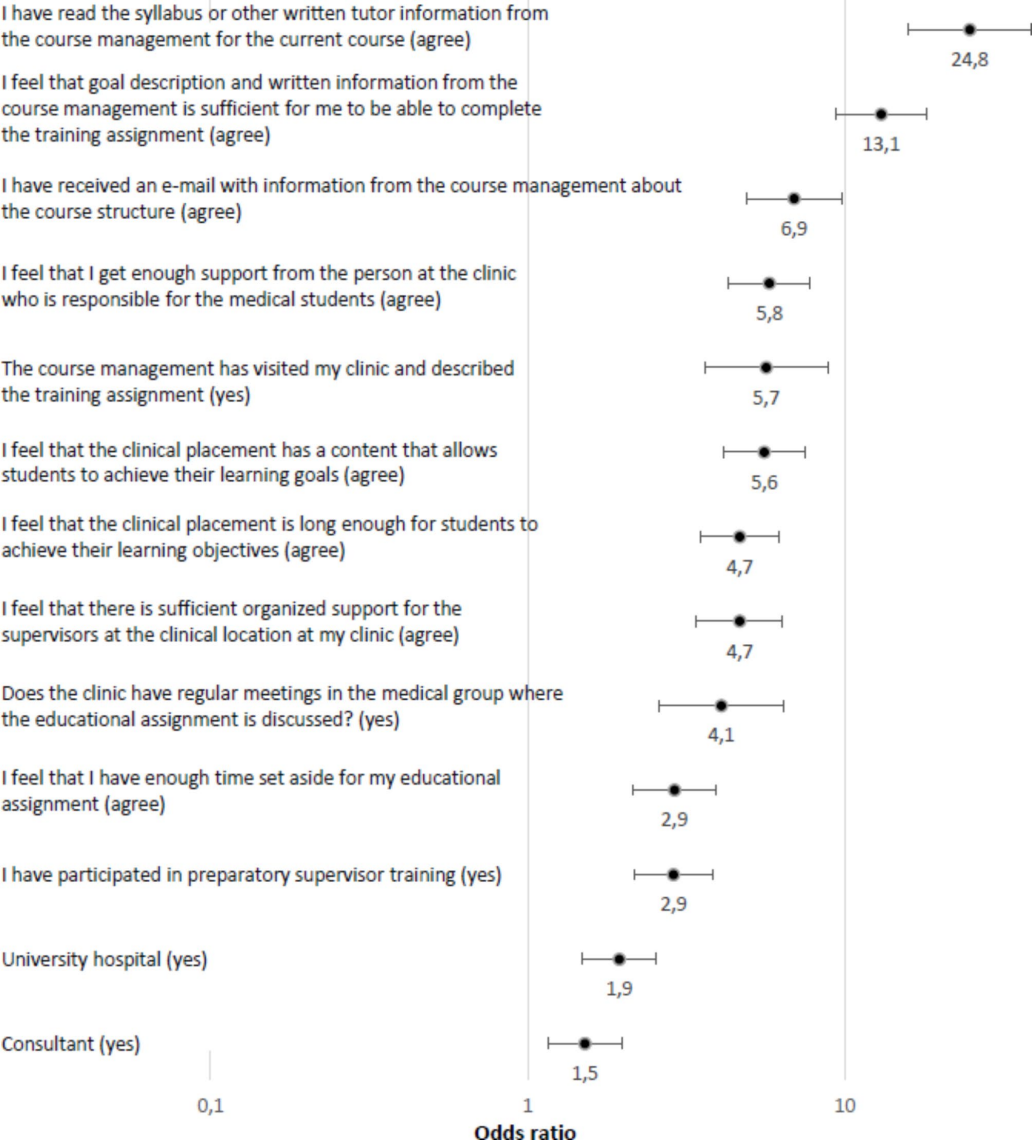
“I am familiar with the current learning objectives of the clinical placement.” Odds ratios and 95% confidence intervals for variables with respondents reporting agree to the statement. Odds ratios with factor present (yes/agree) compared to factor not present, ranked by strength of association

All investigated factors showed significant association with both outcomes chosen as measures for supervisors’ preparedness to teach (Figs. 1 and 2). For the outcome “I feel that I have enough pedagogical knowledge to carry out my educational assignment”, the question “I feel that goal description and written information from the course management is sufficient for me to be able to complete the training assignment” showed the highest odds ratio (OR 7.7 (95% CI 5.4–11.0), followed by “I am familiar with the current learning objectives of the clinical placement” (OR 5.8 (95% CI 4.2–8.1) (Fig. 1). For the outcome “I am familiar with the current learning objectives of the clinical placement”, the question “I have read the syllabus or other written tutor information from the course management for the current course” showed the highest odds ratio (OR 24.8 (95% CI 15.9–38.8) followed by “I feel that goal description and written information from the course management is sufficient for me to be able to complete the training assignment” (OR 13.1 (95% CI 9.4–18.2) (Fig. 2).

## Discussion

In this quantitative survey with responses from clinical supervisors at university and non-university hospitals, we found that most supervisors felt that they were prepared for their teaching assignment. However, one third of the respondents reported that they were not familiar with the learning objectives. We found consistent differences between supervisors at university and non-university hospitals with respect to preparatory supervisor training, knowledge of the training assignment and support.

We confirm findings from the same context in previous qualitative research that several facilitating factors are associated with supervisors self-perceived readiness to teach [27–30]. Especially interaction between faculty and supervisors, through visits to the workplace or emails, and local support to the supervisors showed high odds ratios both for reported knowledge of learning objectives and self-perceived pedagogical knowledge.

### General findings

Although eight of ten respondents reported that they felt that they had enough pedagogical knowledge to carry out their educational assignment, only four in ten had participated in preparatory supervisor training. Consistent with previous findings from qualitative studies, we found a lower degree of participation in supervisor training for clinicians at non-university hospitals [12]. To some extent this difference can be explained by university hospitals having a significantly higher percentage of consultants compared to non-university hospitals. Concerns have been raised in previous research that supervisors without supervisor training tend to rely on various personal teaching methods and lack clear methods for evaluation [34].

Despite seven of ten respondents reporting that they were familiar with the learning objectives, less than half of respondents reported that they had read the syllabus or other written tutor information from the course management. It is possible that some of the supervisors who believe they are familiar with the learning objectives despite not having read them overestimate their understanding. Supervisors not knowing what the learning objectives are for the clinical rotation where they are supervising has been reported by students in previous research [35]. As the syllabus and learning objectives are central in how course management can influence what and how the students are taught, it is important that they are well known by the clinical supervisors [20, 21]. This finding suggests that further efforts need to be made to ensure that all supervisors are aware of the learning objectives.

### Factors that support clinicians to be prepared as clinical supervisors for medical students

Our results identified a variety of factors that associated with supervisors’ preparedness to teach. Supervisors that had received visits from course management and/or emails, were more likely to agree that they had enough pedagogical knowledge to carry out their educational assignment. These supervisors also reported that they were familiar with the learning objectives. From a faculty perspective the findings suggest that interaction between faculty and supervisors is crucial. It follows that recurring visits from faculty used in some decentralized medical schools can be an effective way to support the supervisors [36]. In addition, emails sent to all supervisors each semester can be a cost-effective strategy to increase the supervisors’ knowledge of learning objectives and confidence to teach.

Taking the perspective of the hospital unit as a clinical learning environment presents another viewpoint [37, 38]. Several organizational aspects were associated with the supervisors’ preparedness to teach. Support from the person at the clinic responsible for the medical students, organized support for supervisors along with regular teacher meetings were all associated with supervisors´ reports of being prepared. This highlights the need to develop local communities of practice where education is in focus [16, 39]. Members of the faculty and individuals in the hospital setting dedicated to education, sometimes called local champions, can help bridge the gap between academy and healthcare [40, 41]. Efforts to empower and support the champions enables them in turn to support the clinical supervisors.

Preparatory supervisor training was associated both with self-assessed pedagogical knowledge and familiarity with learning objectives. However, it had a lower odds ratio than interaction with faculty through emails and visits. The finding suggests that supervisor training requires more focus in the specific context where the supervisor is involved and needs to include details on the learning objectives.

### Clinical workload

Most supervisors did not get a reduced clinical workload when they were supervising students. A majority felt that they did not get enough time set aside for their educational assignment. Lack of time to teach is a returning theme in previous studies of clinical supervisors teaching experience and can potentially impact the students learning opportunities [9, 10, 28, 29, 42, 43]. Our finding highlights the constant conflict of interest where training of students often competes with a primary objective of providing healthcare to the patients. As time constraints most likely will remain a challenge to clinical supervision, time management skills are crucial for the supervisors. There are examples of scheduling patients in out-patient care in a way that allows a supervisor to manage two students without a reduction in number of patients seen [44, 45]. Pedagogical models using entrusted professional activities, where students gradually are entrusted to perform tasks in the clinical environment can also serve as a way to shift some of the clinical workload from the supervisor to the student [46–48]. Strategies that can facilitate integration of clinical supervision in clinical practise without a need to reduce clinical workload are crucial to establish.

### Content of clinical placements

Surprisingly, less than a third of respondents reported that they felt that the clinical placement had a content that allowed students to achieve their learning goals and only four of ten reported that they found the clinical placement long enough for students to achieve their learning objectives. This result may reflect that those supervisors are not familiar with the actual learning objectives of the clinical placement. However, it may also reflect a mismatch between expectations from the faculty and the actual learning opportunities the clinical learning environment can provide. From a constructive alignment perspective, it illustrates a challenge in course design where learning objectives need to align with the students learning activities [20, 21]. The findings suggest that clinical supervisors need to understand the learning objectives and more importantly how the objectives can be achieved during the clinical rotation.

### Differences between university and non-university hospitals in how clinical supervisors are prepared for their task

We found supervisors working at university hospitals generally rating slightly higher on the items investigating how they were prepared to teach. Significant differences could be seen when it came to course faculty’s interaction with the supervisors. Part of the difference may be explained by a higher fraction of supervisors being specialists in the university hospital. The findings indicate that more efforts are needed to ensure that supervisors at non-university hospitals are familiar with the learning objectives. Interestingly, at the non-university hospitals, there was a significantly higher number of supervisors who reported a reduction in clinical assignments during periods with educational assignments.

### Strengths and limitations

This study was distributed to all clinical supervisors supervising medical students from a university with clinical rotation at multiple hospitals. It adds new knowledge on how clinical supervisors are prepared for their teaching assignment and allows comparison between supervisors at university and non-university hospitals teaching students from the same university. Our results are limited to the educational context of a Swedish university with regionalised medical education. However, the results conform with previous qualitative research which indicates that it can be used to better prepare clinical supervisors working under similar conditions at other universities. Our study has some further limitations. The response rate to the survey was 43%, making it difficult to say with certainty that the results apply to the whole population. There is also a need to be aware that this a cross sectional study, and associations should be interpreted with caution as we cannot make inferences with respect to causality. Finally, self-perceived preparedness is not a perfect measure of actual ability to teach and further studies investigating the supervisors’ actual abilities and knowledge of learning objectives is needed to confirm our findings.

## Conclusions

In conclusion we found that supervisors’ self-perceived preparedness was associated with familiarity with the learning objectives, faculty involvement through visits to their workplace and emails as well as local organizational support. We found consistent differences between supervisors at university and non-university hospitals with respect to preparatory supervisor training, knowledge of the training assignment and support.

This implicates that when students are sent to non-university hospitals, it is crucial to ensure that the new clinical supervisors are prepared for their assignment. In this process, communicating the learning objectives is central.

## Data Availability

The datasets used and/or analyzed during the current study are available from the corresponding author in response to reasonable requests.
